# Differential dementia detection from multimodal brain images in a real‐world dataset

**DOI:** 10.1002/alz.70362

**Published:** 2025-07-01

**Authors:** Matthew Leming, Hyungsoon Im

**Affiliations:** ^1^ Center for Systems Biology Massachusetts General Hospital Boston USA; ^2^ Massachusetts Alzheimer's Disease Research Center Massachusetts General Hospital Boston USA; ^3^ Department of Radiology Massachusetts General Hospital Boston USA

**Keywords:** AI, biomarkers, deep learning, dementia diagnostics, electronic health records, MRI, multimodal

## Abstract

**INTRODUCTION:**

Artificial intelligence (AI) models have been applied to differential dementia detection tasks in brain images from curated, high‐quality benchmark databases, but not real‐world data in hospitals.

**METHODS:**

We describe a deep learning model specially trained for disease detection in heterogeneous clinical images from electronic health records without focusing on confounding factors. It encodes up to 14 multimodal images, alongside age and demographics, and outputs the likelihood of vascular dementia, Alzheimer's, Lewy body dementia, Pick's disease, mild cognitive impairment, and unspecified dementia. We use data from Massachusetts General Hospital (183,018 images from 11,015 patients) for training and external data (125,493 images from 6,662 patients) for testing.

**RESULTS:**

Performance ranged between 0.82 and 0.94 area under the curve (AUC) on data from 1003 sites.

**DISCUSSION:**

Analysis shows that the model focused on subcortical brain structures as the basis for its decisions. By detecting biomarkers in real‐world data, the presented techniques will help with clinical translation of disease detection AI.

**Highlights:**

- Our artificial intelligence (AI) model can detect neurodegenerative disorders in brain imaging electronic health record (EHR) data.
- It encodes up to 14 brain images and text information from a single patient's EHR.
- Attention maps show that the model focuses on subcortical brain structures.
- Performance ranged from 0.82 to 0.94 area under the curve (AUC) on data from 1003 external sites.

## 1 BACKGROUND

The use of artificial intelligence (AI) models to classify brain images for disease detection is often repeated on a growing number of public benchmark datasets,^1^, ^2^, ^3^, ^4^ but remarkably little of this work has translated to the clinic. The reasons for this untranslatability are complex and include institutional barriers, technical challenges, and, for certain diseases, insufficient biomarkers in patient data.^5^ Lack of widespread availability of real‐world clinical data to most researchers slows progress on the many challenges of applying deep learning to confounded and imbalanced real‐world data,^6^ though the availability of anonymized patient data in certain research hospitals has led to steady progress. A growing number of large‐language‐model‐based AI methods have been released to make real‐world predictions from text data in electronic health records (EHRs),^7^, ^8^, ^9^, ^10^ while other work in deep learning for clinical images has been undertaken in recent years on preprocessing tasks.^11^, ^12^ These developments show the technical feasibility of applying AI to medical images in EHRs. However, even with these developments, use of this technology for disease detection with highly heterogeneous clinical imaging remains understudied. This is partially due to the added complexity of working with imaging data, not only because it requires additional expertise to encode into AI models but because it also carries increased risk of machine learning bias.^6^ To list a few examples of this bias: age bias, in which a model would focus unduly on age‐related biomarkers, complicates the detection of rare early‐onset cases of neurodegenerative diseases, and it is a challenge to directly regress effects of age‐related biological variables (e.g., head size) from imaging data; similarly, analysis of fluorodeoxyglucose‐positron emission tomography (FDG‐PET) images, a modality that is commonly acquired in memory clinics,^13^ would suggest by its mere presence that the patient has memory impairment. Ideally, we would like to ensure that a model focuses on true disease‐related biomarkers, such as cortical thinning or white matter degeneration, in its disease detection decision when using the information present in a brain image.

**RESEARCH IN CONTEXT**

1. **Systematic review**: The authors reviewed the literature using traditional sources (medRxiv, PubMed). While disease detection studies using brain imaging data are common, the application of artificial intelligence (AI) to real‐world heterogeneous brain imaging data at a large scale is rare. Some recent work has utilized text data from electronic health records (EHRs).
2. **Interpretation**: Our primary contribution is the use of routinely collected brain imaging data for disease detection, as well as our evidence suggesting that this is largely done by analysis of biomarkers in the imaging data rather than technical confounds that are correlated with the presence of a disease. This work is necessary for the clinical translation of such models towards deployment and day‐to‐day clinical use.
3. **Future directions**: Two potent future directions are studies on large datasets and methods to develop explainable AI for neuroimaging disease detection. Another is the application of this work to prognostics and outcome predictions rather than differential detection.

While a challenge, recently developed computer vision methods make working with such complex data possible. In this work, we present a deep learning model trained using a number of novel regression techniques that can mitigate such bias. Our model can encode multiple pieces of text and imaging data from a single patient's EHR and output predictions of the presence of five different neurodegenerative disorders commonly known to cause dementia.^14^ Our model is not only useful clinically for disease detection but also for studying such diseases from the very large datasets present in hospital data archives. Given the unique internal resources of Mass General Brigham, including a vast archive of brain images in EHRs – from Massachusetts General Hospital (MGH), Brigham and Women's Hospital (BWH), and data imported from different hospitals in and out of Massachusetts – we are able to apply methods long‐studied on public benchmark data to routinely collected medical imaging data in hospital archives. We also present a further analysis of the model's attention maps, which suggests that it works by analyzing imaging biomarkers, as well as the comorbidities in the patient population that substantively affected the model's performance.

## 2 METHODS

### 2.1 Data

This is a case–control study on anonymized archival data from MGH and BWH internal archives, collected between 1995 and 2021. This work was conducted under Institutional Review Board (IRB) 2015P001915 and was conducted using all of the appropriate institutional ethical standards. This data is largely representative of the population of Greater Boston that had to receive a brain scan at MGH and BWH, though in many cases patients imported their data from outside systems. The training and external testing data are described in Table 1. Our models were trained exclusively on MGH data and tested on BWH and external‐site data, to simulate real‐world site differences. We separated patients first by a broad dementia, non‐dementia, and exclusion label. Patients with a head injury or malignant neoplasm were excluded, and test results between disease and non‐disease data are presented. We further applied labels from the non‐excluded groups into eight separate neuropsychiatric disorders: Alzheimer's, vascular dementia, Lewy body dementia, Pick's disease, mild cognitive impairment (MCI), epilepsy, and multiple sclerosis. Due to the small numbers of patients with Pick's disease, MCI, and Lewy body dementia, we also applied a general classification for neurodegenerative disorders (International Classification of Diseases, revision 10 [ICD‐10] code G31).

**TABLE 1:** Description of the patients and disease groups between the training and test set.

Patient group		MGH (train)					BWH/external (test)				
Desc.	ICD	No. of images	No. of patients	Age (avg ±std)	No. of male	No. of sites	No. of images	No. of patients	Age (avg±std)	No. of male	No. of sites
Dementia	‐	54428	2734	63.7±14.7	1407	49	31959	1504	60.0±14.8	696	221
Non‐dem.	‐	128590	8281	38.6±16.9	3881	62	70514	5158	42.2±15.2	2148	473
Exclude	‐	82050	3857	55.3±16.4	1947	64	93534	3917	54.6±14.1	1769	552
Vasc. dem	F01	1388	93	73.6±8.5	43	18	937	58	71.1±9.9	32	30
Alzheim.	G30	3422	265	71.3±7.9	131	24	1941	154	71.8±7.8	75	37
Neurodeg. diseases	G31	5198	369	70.7±9.0	189	24	3017	212	70.4±9.4	102	60
Pick's	G31.01	255	24	72.3±6.0	14	15	113	9	69.7±7.8	5	7
Lewy body	G31.83	396	35	71.3±7.2	26	15	402	26	73.0±5.0	14	19
MCI	G31.84	3591	264	71.6±8.0	130	23	2252	162	72.4±7.1	73	53
Parkin.	G20	2640	177	69.8±9.4	122	22	866	63	70.8±9.5	41	27
M.S.	G35	3839	160	46.7±13.6	65	24	12974	453	48.2±13.2	139	180
Epilepsy	G40	6583	223	48.6±17.2	121	30	5245	210	51.2±17.0	105	88

*Note*: The wider dementia group (top) was determined with a combination of ICD codes and medication history.

Abbreviations: Alzheim., Alzheimer's disease; BWH, Brigham and Women's Hospital; ICD, International Classification of Diseases; MCI, mild cognitive impairment; MGH, Massachusetts General Hospital; M.S., multiple sclerosis; Parkin., Parkinson's disease; Vasc. dem, vascular dementia.

The general “dementia” label was applied to patients with ICD‐9 or ICD‐10 codes indicating a neurodegenerative disorder, or a previous prescription of memory impairment drugs (galantamine, memantine, donepezil, rivastigmine). It was developed to envelop as broad a population as possible from our dataset, though as a label it is weaker than the others applied. The additional neuropsychiatric diseases were also identified based only on ICD‐10 codes (or ICD‐9 translated into 10) of various neuropsychiatric disorders (Table 1).

**FIGURE 1:**
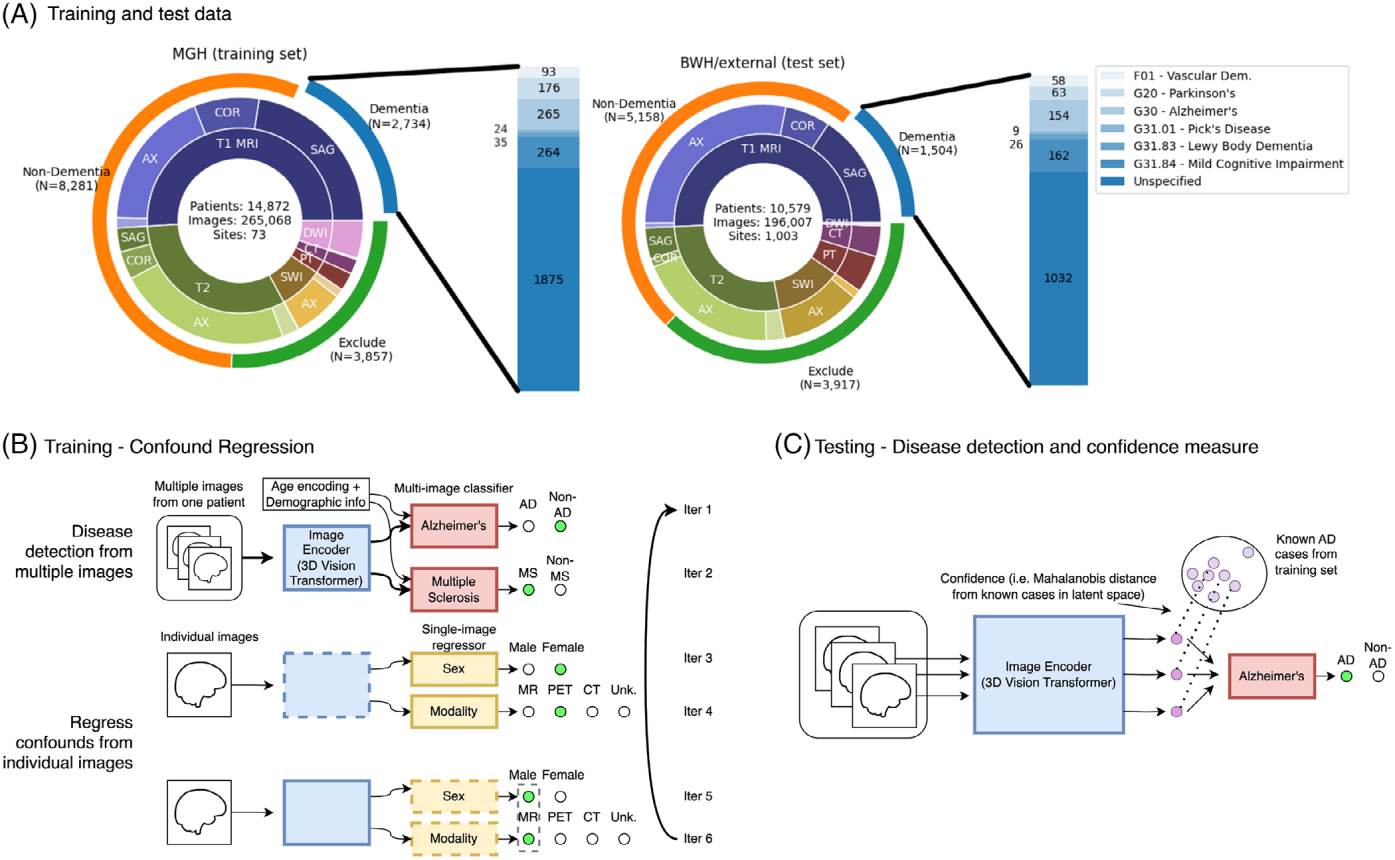
A diagram of the data and the multi‐ and optional‐input deep learning model. (A) A description of the training and testing cohort (see also Table 1). (B) The image encoder is trained first with multiple brain images, mapping each to a latent space, as well as age and demographic information, to classify by a specific disease type. The encoder is further trained adversarially to remove any information about confounding factors by making the latent space appear to be the most frequent representation of each image (e.g., the latent representation of positron emission tomography [PET] images is made to look like magnetic resonance [MR] images). (C) During testing, the Mahalanobis distance of each test image is measured between that image and the distribution of latent vectors of its predicted label in the training set. This can be used as a confidence measure to detect out‐of‐distribution images.

Figure 1A shows the patient‐wide distribution of our training and test data, as well as a distribution of the imaging modalities in each set. The data are imbalanced, particularly by age, though this is accounted for during training by sampling patients from the same age distribution in a data matching scheme.

### 2.2 Multi‐ and optional‐input framework with confound regression

Figure 1B,C shows the architecture and training/testing schema of our model, which can take in between 1 and 14 brain images, as well as text input about ethnicity and sex. Images are encoded with a 3D vision transformer^15^ to a 1×128 latent representation, and text data are hashed to a 1×128 vector (note that we are treating text data as categorical tokens rather than words, so more complex embeddings such as Word2Vec are not necessary). These vectors are then stacked to a consistent 16×128 array, with positional encoding added to indicate patient age at the time of the scan.

The image encoder uses an adversarial method^16^, ^17^ that builds on our previous work^18^ to mask the effects of confounds, such as sex, imaging modality, age, and site (Figure 1B). It trains a regressor to detect each of these confounds from the 1×128 latent representation of each image, while the encoder is trained to map each label to the most‐represented label in the training set – for instance, making computed tomography (CT) and PET image representation look like an MR image and female brains look like male brains – which makes each confound‐based grouping indistinguishable to the classifier. We directly encoded patient age and sex, as well as the modality, angle, slice thickness, and scanning sequence of the input image, as confounds to be regressed during training. Data were additionally matched by patient age post‐hoc to further mitigate ML bias. This matching was performed multiple times during training; an age‐matched batch would be sampled and fed to the model, then another would be resampled. In this way, no data were explicitly excluded from training, even those on both ends of the age spectrum, though some were more highly represented. The training scheme for this model closely resembles the training scheme of Generative Adversarial Networks (GANs)^19^; thus, we were able to utilize the wide amount of literature on best training practices for GANs when fine‐tuning these models.

**FIGURE 2:**
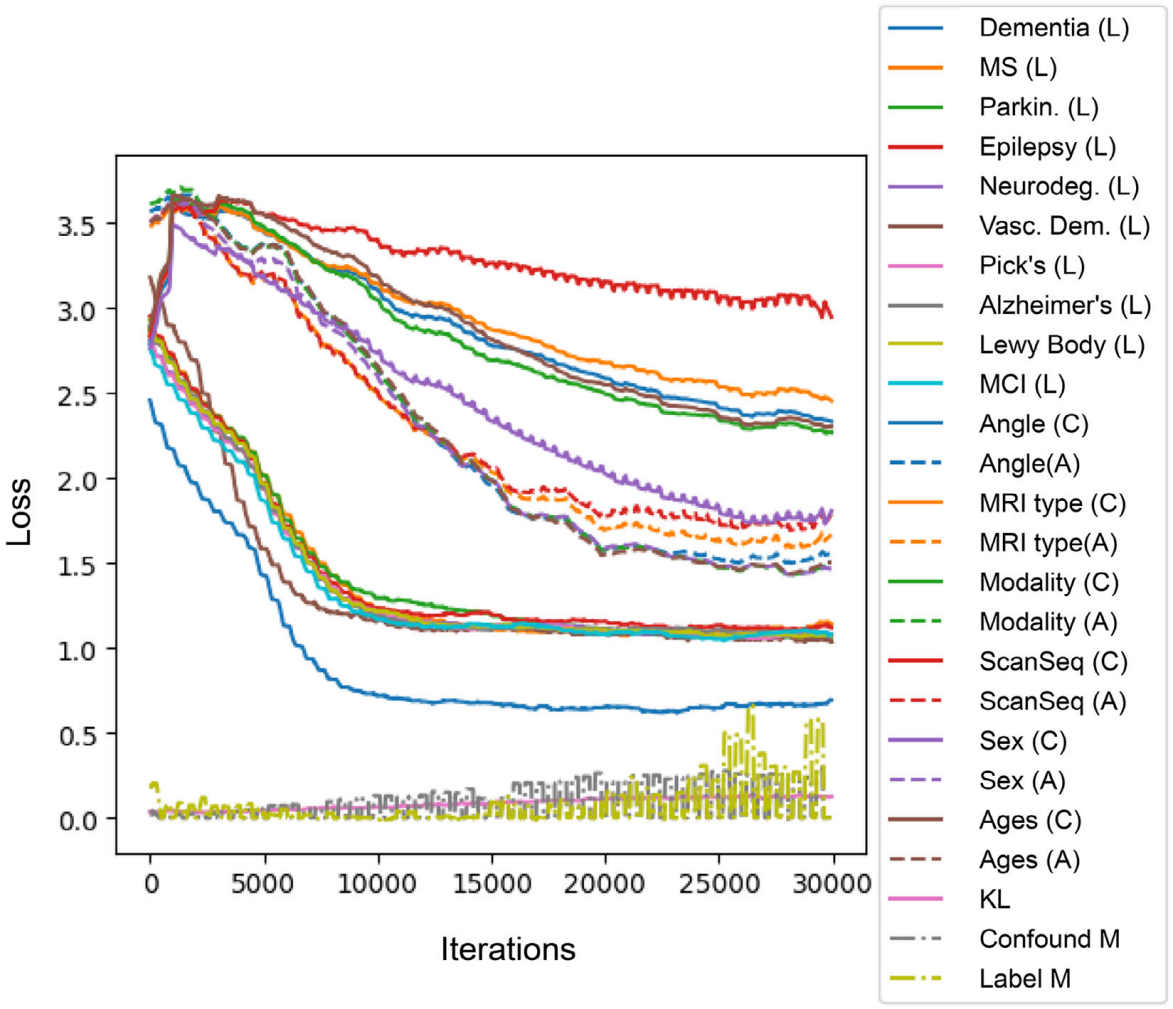
The training of the model and the different loss functions involved. *L* indicates the labels trained for; *C* and *A* indicate the adversarial confounds trained for. Also shown is the Kullback–Leibler divergence loss, as well as the clustering and de‐clustering losses.

To further mitigate against potential bias, we implemented several additional loss functions, as shown in Figure 2. One loss function incentivized clustering in the latent space by a given disease label (“Label M” in Figure 2) and de‐clustering (“Confound M”) by a given confound label. This was implemented as follows:

$$loss\left(I\right)=\left(I-\mu_{L=l}\right)-\left(I-\mu_{L\;l}\right)+\left(I-\mu_{C\;c}\right)-\left(I-\mu_{C=c}\right)$$

where *I* is the latent representation of a given image, *μ* is the mean latent vector, l is the label of *I*, *L* is the set of all labels, *c* is the confounds of *I*, and *C* is the set of all confounds. This thus minimized the Euclidean distance between points in the higher‐dimensional latent space that were of the same label while maximizing the distance between the confounds, preventing high‐dimensional clustering by confounding labels.

Finally, we implemented a confidence measure to remove out‐of‐distribution images at test time. We saved latent representations of given disease labels from the training set and measured the Mahalanobis distance between training images and a given input to assess overall confidence^20^ (Figure 1C):

$$C\left(I\right)={\left(I-\mu_{L=l}\right)}^{T}S_{L=l}^{-1}\left(I-\mu_{L=l}\right)$$

Where *S* is the 128 × 128 covariance matrix of the set of training images with a given label and *μ* is the 1×128 mean. This, in effect, allowed us to implement a confidence measure for each individual image and eliminate a number of out‐of‐distribution brain images at test time from consideration. To stabilize the Mahalanobis measurement, the encoder is forced to maintain a Gaussian distribution for its latent outputs, using the same mechanism as a variational autoencoder.^21^

### 2.3 Comparative performance on metadata

While we matched for age during training, our overall population is nonetheless skewed. Thus, even with extensive regression tasks, there is still a possibility that the model may use these imbalances to achieve high performance. To offer insight into the added value of confound‐regressed imaging data in this patient sample, we perform an analogous classification using only text‐based metadata. Essentially, we input the confounding variables themselves into a machine learning model and assess the performance from that. In this task, we input age, sex, ethnicity, and imaging metadata (modality, angle, slice thickness, and scanning sequence) without the images themselves, to achieve a per‐image classification of each task. A random forest model is used for this test, due its general ability to outperform deep learning on tabular data.^22^ This shows how well the models could perform using only confounds, and thus what the added value is, in theory, of using a model that takes into account biological information present in medical images.

### 2.4 Technical factors and comorbidities affecting model performance

Our model is capable of using information from multiple brain images of differing modalities. To test the effects of different imaging modalities on model performance, we randomly input different combinations of images from patient's EHR, grouping them together (e.g., isolating a subset of all patients with at least one DWI and at least one PET scan in the model, or all patients with between 6 to 10 images), and measuring the AUC of each grouping.

We additionally analyze which comorbidities affect model performance by analyzing patient ICD‐10 codes. This is achieved by comparing, for each patient, the average model performance (measured by 1 ‐ |Y ‐ Y'| – i.e., the error of the prediction); the dependence of the comorbidity on model performance (i.e., whether or not the model made better or worse predictions on patients with acute hypertension); and the dependence of the comorbidity on the presence of the disorder itself (i.e., whether or not more patients than average with acute hypertension also had dementia). We used the Kruskal–Wallace test to measure a statistic indicating the dependence of the model prediction error (1 ‐ |Y ‐ Y'|) on the presence of the disorder. The dependence of comorbidities to the disease label being predicted was determined using the Chi‐Squared dependence test. This allows us to assess which comorbidities affected model performance with the highest statistical significance.

### 2.5 Analysis of attention maps

3D vision transformers output attention maps that show where the model focused when making its classification decision. They are designed to do this for single images. To quantify which brain areas the model focuses on in its classification, we pass a minimum of 20 samples of brain images with each neurological disorder through a SynthSeg^12^ parcellation. We then applied the mapping of the attention maps from our transformer and to these parcellations and averaged them. They are then normalized into a z‐score, and the average attention in each area of the parcellation are further averaged. This shows the areas of the brain images that the model focused on when making a disease detection decision, thus also showing which areas of the brain are most associated with each neurological disorder. We show which areas of the brain the model focused on at least one standard deviation above average (i.e., the 68th percentile) on the samples.

## 3 RESULTS

### 3.1 Overall model performance on disease detection tasks

**FIGURE 3:**
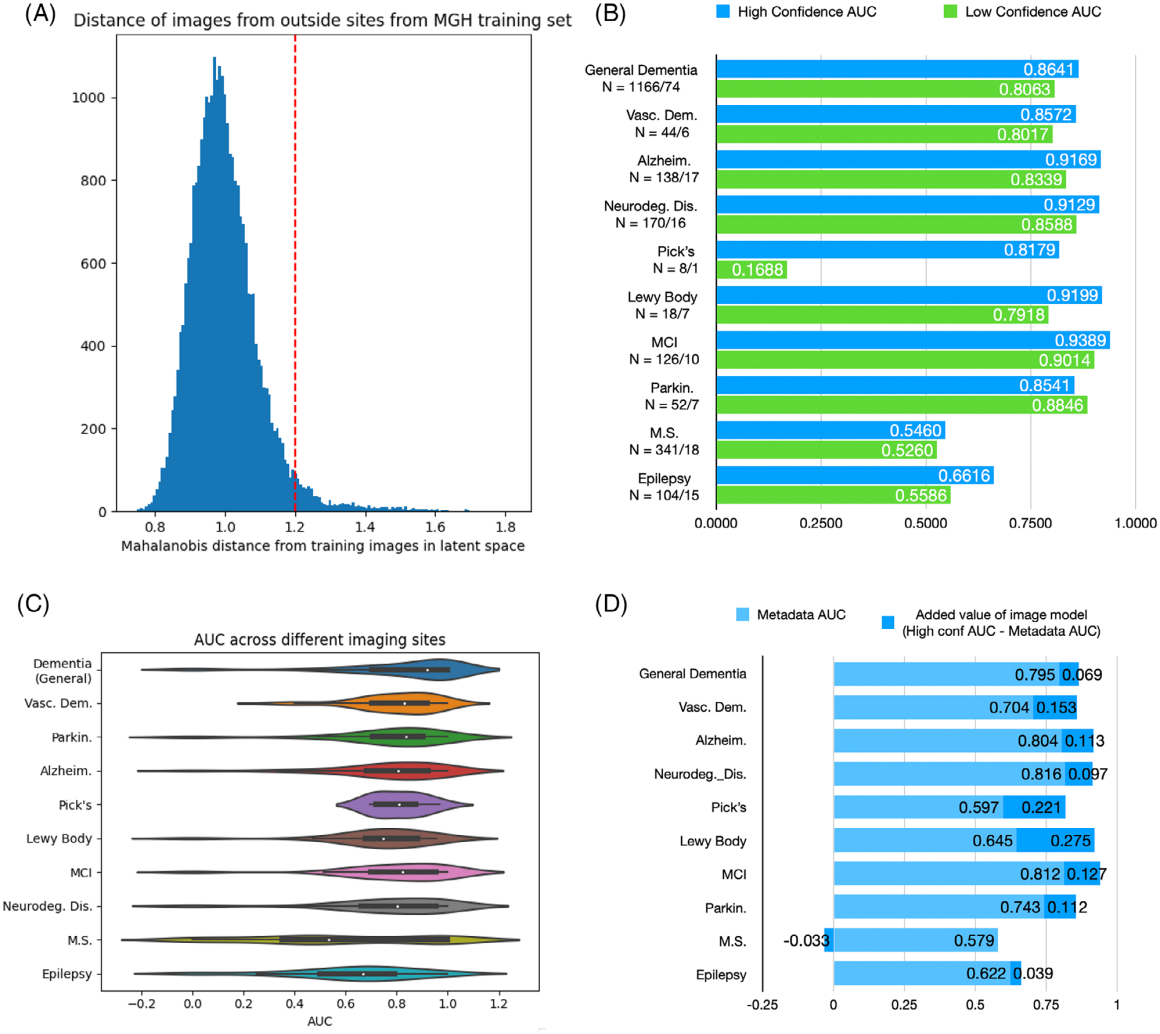
A description of the overall performance of the model. (A) Distribution of the Mahalanobis distances in latent space between the external site imaging data and the training set. This clearly shows two distributions, marked by the *x* = 1.2 line (in red), indicating a separation between in‐distribution and out‐of‐distribution data. (B) The areas under the curve (AUCs) for each disease measured on the in‐distribution dataset (blue) and out‐of‐distribution dataset (green), showing a substantial improvement in performance in each case. In grey, the AUC when using only non‐imaging data for the classification is shown, offering a baseline for how much the model offers in predictive value. (C) Variation of performance across different external sites. (D) The difference between the high‐confidence predictions and the predictions from the metadata of the images alone, offering an approximation of the added value of imaging information for each task.

Figure 3 shows the overall model performance on each of the disease detection tasks, on both the in‐distribution and out‐of‐distribution data. Figure 3A shows the distribution of the confidence measures of each input image for the general, dementia‐versus‐non‐dementia task. Most images in the test set fell into a normal distribution, but a few trailed; these are separated as out‐of‐distribution (i.e., “low confidence”) datapoints by the dotted red line. Figure 3B shows that the model performed well when classifying the neurodegenerative disorders, with a minimum of 0.82 AUC (Pick's disease) and maximum 0.94 AUC (MCI). Our model gave close to random performance when classifying multiple sclerosis and epilepsy. We additionally classified by neurodegenerative disorders generally – ICD code G31 – which is a label that includes patients with MCI, Lewy body dementia, and Pick's disease.

As shown in Figure 3C, the model varied across tasks in its cross‐site performance, even though the average was favorable. In general, this shows that the model performs well on external site data. Nearly all cases in which the model exhibited low performance were those with a relatively small number of images, with performance being normalized on sites that constituted a larger portion of the test set (see Figure S1). We additionally performed an analysis on images from the first patient visit, which shows that this model would be effective in detecting diseases upon an initial screening (see Figure S2).

### 3.2 Comparative performance on metadata

The results of the comparative metadata task, in which we compare the high‐confidence predictions in 3B with the machine learning model that classified the same data using only confounding variables as inputs, are shown in Figure 3D. This test effectively shows how much information in the imaging data increases disease detection performance compared to just text data in the EHR itself. Notably, the model showed significant additive value when detecting Lewy body dementia (0.275), vascular dementia (0.154), and Pick's disease (0.221).

### 3.3 Technical factors and comorbidities affecting model performance

Because the model accepted multiple images for a single prediction, and many patients have multiple types of images in their EHRs, we tested, for each patient, different combinations of images. This helped us to isolate a sample of which types of data were most effective and caused the largest overall uptick in model performance.

**FIGURE 4:**
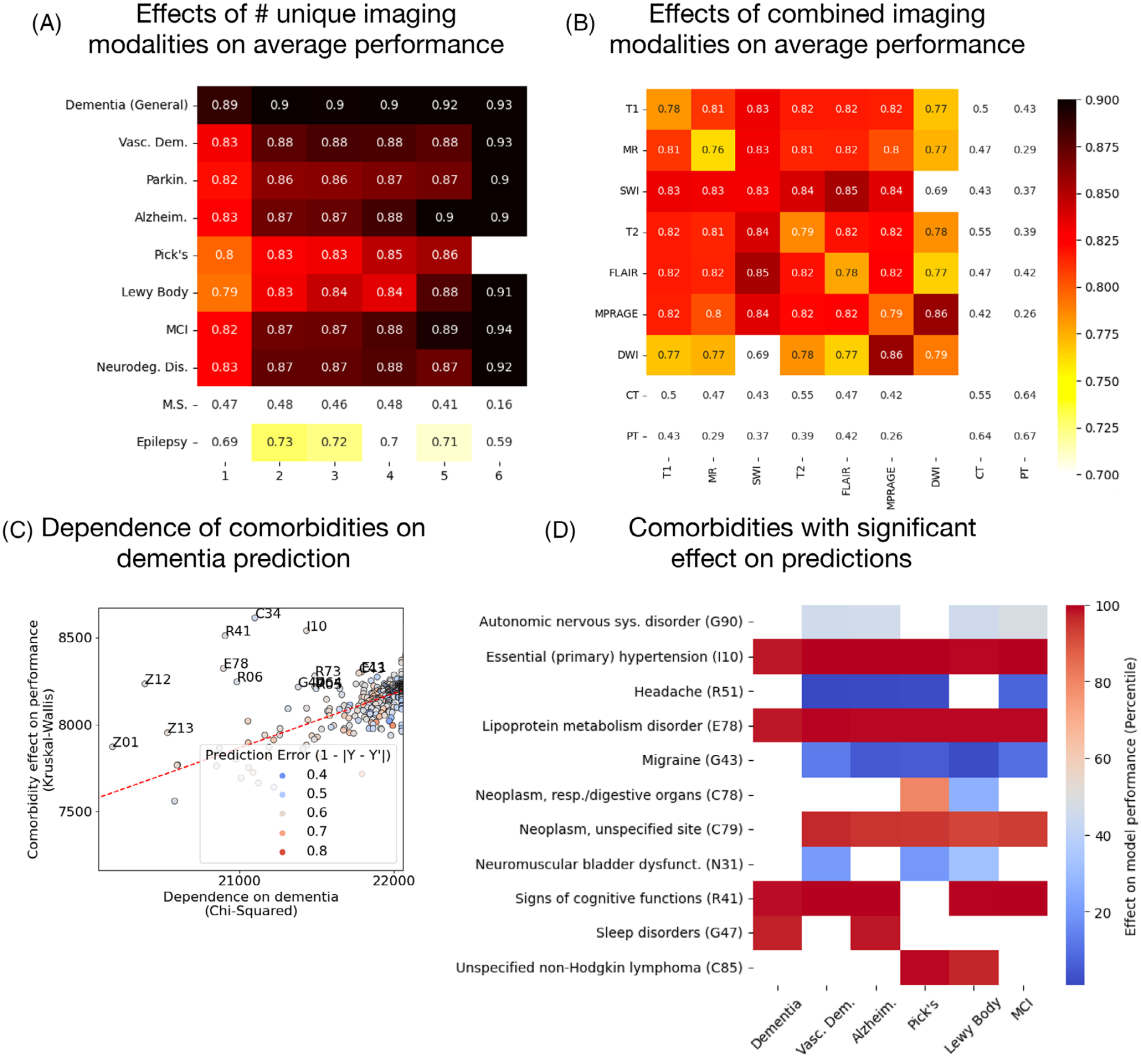
Factors that affected model performance. (A) The performance, across all tasks, when multiple imaging modalities and multiple images are input into the model, showing that the performance rises the more types of information are present. (B) The performance when different combinations of modalities are input. The middle diagonal indicates the performance when only a single imaging modality is input. (C) Comorbidities that uniquely affect model classification. The correlation between the effect of a comorbidity on the model's ability to classify general dementia and the contingency of general dementia on those ICD codes. (D) The comorbidities that affect model performance the most, with blue indicating that it caused the model to underperform and red causing it to over perform. These comorbidities were selected in a way that accounted for their dependence on the disease being classified for, i.e., the standout datapoints above the regression line in part C.

Figure 4 describes the different factors in our data that affected model performance. Figure 4A shows the increase in model performance when more different types of imaging modalities are input into the model. Figure 4B shows the performance when different MRI modalities shows the performance when individual imaging modalities are input into the model alone, including seven types of MRI as well as CT and PET. CT and PET drastically underperformed compared to MRI modalities. This was likely due to the adversarial regression techniques, which disincentivized the model from using imaging modalities explicitly in its classification decision; because relatively few patients had CT and PET in their EHR alongside MRI, the presence of PET and CT likely indicated the presence of one or more neurological diseases. The low representation of PET and CT in the training set (Figure 1A) also likely contributed. This would have effectively caused the model to lower its own performance since such confounds could not be disentangled from the disease biomarkers of interest. Notably, as well, the inclusion of susceptibility‐weighted images (SWI) provided slightly higher AUC, suggesting that visualization of blood vesicles aided in disease detection (this is supported by the comorbidity results described below). These tests established several intuitive results, namely that more data, and more types of data, input into the model leads to higher performance.

Figure 4C,D shows the comorbidities, as indicated by ICD codes, that affected model performance with any statistical significance. The two disorders that that led to increased performance in detection of all neurodegenerative disorders were essential hypertension and lipoprotein metabolism disorder, two known risk factors for dementia.^23^, ^24^ The over‐performance when SWI is input, combined with the over performance in the presence of essential hypertension, strongly suggests that the model focused on blood vesicles in many of its decisions. Interestingly, the presence of headaches, migraines, and bladder dysfunction led to underperformance of the model; this may indicate the misdiagnosis of neurodegenerative disorders based on outward symptoms. The presence of other psychiatric disorders, namely, sleep disorders (G47), a common symptom of Alzheimer's,^25^ and different cognitive function disorders (R41, which often indicates different forms of amnesia) added model performance in most tasks. Other comorbidities are likely to be purely associative: for example, the presence of neoplasms and non‐Hodgkin lymphoma helped the model predict the presence of Pick's and Lewy body dementia, which is in line with studies linking dementia risk with risk of cancer.^26^, ^27^

### 3.4 Attention maps

**FIGURE 5:**
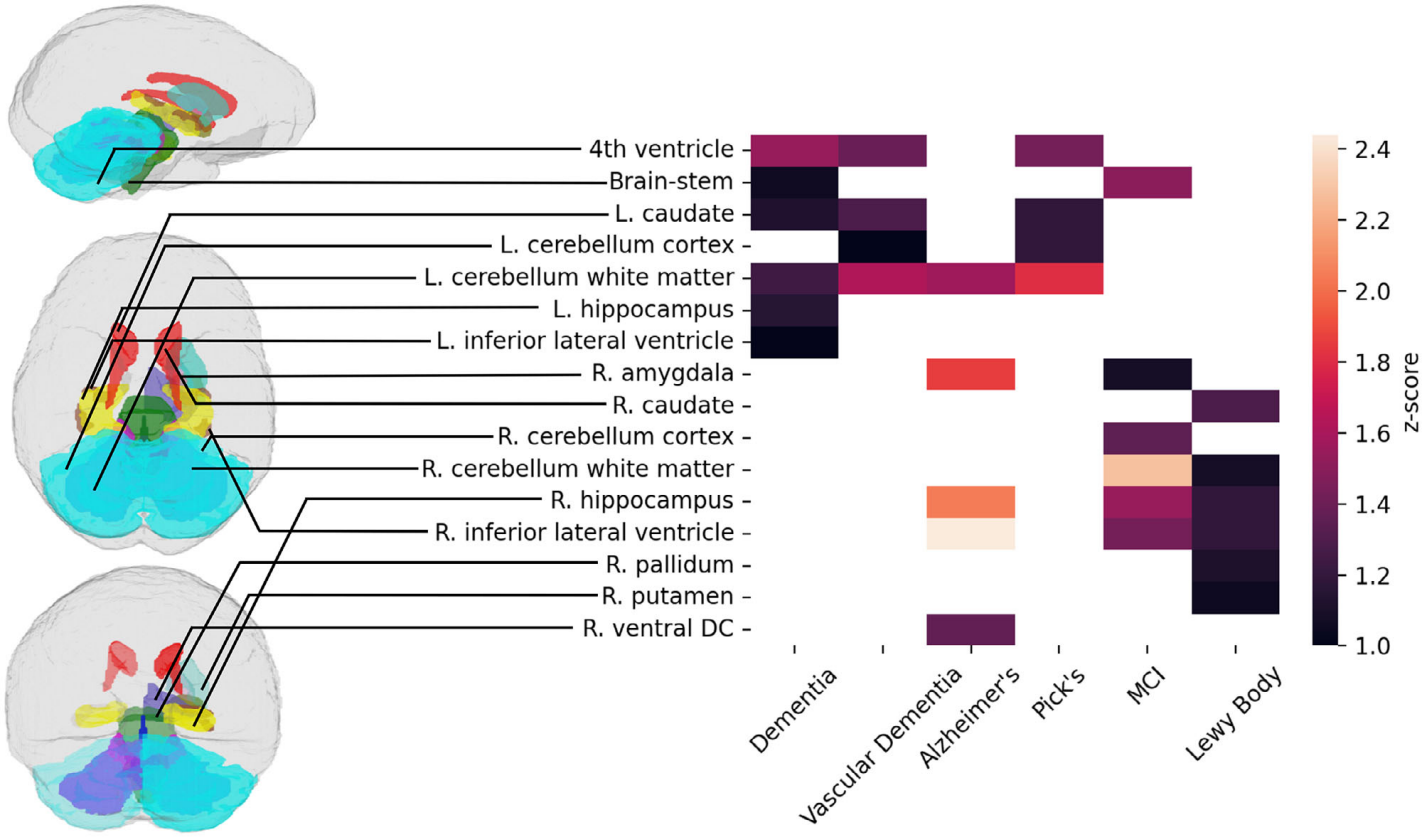
Areas that the model focused on the most for each neurodegenerative classification task, projected onto a SynthSeg parcellation. Our model's attention maps tended to focus on the relative sizes of subcortical brain structures for its differential detections, many of which have been shown in previous studies to be associated with neurodegenerative disorders. Brain structures are colored for visual effect.

Figure 5 shows the average focus of multiple attention maps for each disease analyzed. The model focused on 16 brain areas with statistical significance: the fourth ventricle, the brain stem, the bilateral caudate, bilateral cerebellar cortex, bilateral white matter of the cerebellum, the bilateral hippocampus, the bilateral inferior lateral ventricle, the right amygdala, the right pallidum, the right putamen, and the right ventral diencephalon. Five of these (left hippocampus, left inferior lateral ventricle, right ventral diencephalon, right cerebellum cortex, and right caudate) were the area of focus for only one disease, while the rest were the focus of multiple diseases.

The model focused on areas of the left hemisphere for all‐cause dementia, particularly the left hippocampus and left inferior lateral ventricle, even while it focused on the right hemisphere for Alzheimer's, MCI, and Lewy body dementia. There are a few possible reasons for this, though it's difficult to tell whether they're based in neurobiology or data imbalances or a combination. This may be an indication of other, unidentified subgroups in the general dementia group that are not fully explained by the other labels. It may also indicate the relative severity of changes Alzheimer's, MCI, and Lewy body have in areas of the right hemisphere, while structures in the left hemisphere were more affected on average across dementia group as a whole.

These are all subcortical areas, which have been generally associated in previous studies with the presence of neuropsychiatric disorders. The Rotterdam study, for instance, found that subcortical structures were highly associated with dementia risk.^28^ Multiple studies have noted the enlargement of the lateral ventricles in neurological diseases.^29^, ^30^ Lewy Body dementia, which this model was particularly successful at detecting, typically manifests as lesions that appear in more localized areas of the brain stem, limbic and neocortical areas,^31^ which this model also focused on. In Alzheimer's disease and MCI, the focus on the inferior lateral ventricle and the hippocampus is strongly reminiscent of previous research that has found shrinkage in the hippocampus and enlargement of the lateral ventricle.^32^ Shrinkage in cerebellar white matter has also previously been associated with MCI.^33^

The overall poor classification of the comparison tasks (multiple sclerosis and epilepsy) would suggest that the model prioritized focus on brain structures associated with neurodegenerative disorders, which constituted the majority of tasks it trained on (Figure 2). Additionally, the biological basis of multiple sclerosis is lesions that may appear in many different areas of the brain,^34^ further suggesting that the model largely made its decisions by analyzing the relative size of subcortical structures rather than detecting brain anomalies that may be scattered in different brain locations.

The model's attention may, additionally, be driven by purely technical considerations as well. For instance, in making its disease detection decision, it tended to shift its focus to individual hemispheres. This may be due to different neurodegenerative disorders affecting one hemisphere uniquely,^35^ or it may be due to the model's tendency to reduce redundancies in its analysis. Subcortical structures are also visually distinct and easier to pick out of a brain image than, for example, localized alterations in cortical grey matter. This is particularly true of their appearance in low‐resolution brain images.

## 4 DISCUSSION

Our model is the first that is capable of fully encoding imaging data from an EHR, effectively addressing the task of incorporating very complex and heterogeneous medical imaging data in automated decision‐making. While many previous studies have made use of one or many public benchmark data sets, very few have made use of routinely‐collected clinical brain imaging data, and this is the first to utilize such data for a differential detection across a range of disorders, offering an output of each disorder simultaneously. This framework is useful not only for potential clinical applications, but also as a toolkit to study the disease‐based information in large‐scale clinical imaging data.

While there are continuing challenges in overcoming the “black box” nature of deep learning models, our analysis strongly suggests that our framework helps to mask technical confounds from consideration in the disease detection process and can incorporate multiple pieces of relevant biological information in its decision‐making process. Our attention map analysis strongly suggests that this model analyzed disease biomarkers in its classification. This is backed up by the superior cross‐site performance across tasks, having superior (> 0.82 AUC, Figure 3) performance in eight tasks, though with random performance in two others.

Our data were highly imbalanced in the age distributions (see Table 1), but the model was designed to use numeric age (which was encoded directly in the classifier; see Figure 1B) while not using biomarkers in brain images purely related to age. During training, patients were matched by age from the MGH dataset, and age was one of the confounds adversarially regressed from the images (Figure 1B). Furthermore, the input of age at a later stage in the classification disincentivized the model from seeking that information directly from the image, as it would have been redundant. Thus, the model was strongly disincentivized from using age‐related biomarkers from the brain images in its disease detection decision, even while it used the numeric age directly. This was done to both boost model performance and simulate the likely usage of real‐world AI systems, which would ideally utilize as much contextual information as possible in their classification.

In this study, the only non‐imaging data we opted to use as model inputs were age, sex, and ethnicity. Other works on EHR analysis included medication history and ICD‐10 codes in their prediction models.^9^ Encoding more text data from EHRs into our models is very possible, but combining these with labels derived from ICD‐10 codes brings up further questions of causality, since our labels relied on the same ICD‐10 codes,^36^, ^37^ and other ICD‐10 codes were correlated with the presence of dementia (Figure 4C). Model performance can likely be improved by adding more such information about patient disease history, but the goal of this study was to make the diagnosis rely on brain images as much as possible. Thus, the model was strongly disincentivized from using age‐related biomarkers from the brain images in its disease detection decision, even while it used the numeric age directly. This was done to both boost model performance and simulate the likely usage of real‐world AI systems, which would ideally utilize as much contextual information as possible in their classification.

During data analysis, we tried to leverage the size of these data to produce meaningful results using unsupervised clustering as well as supervised ML methods, but it was difficult to find any meaningful interpretation of the unsupervised results. Several methods, including deep auto encoders and principal component analysis, were employed to reduce the dimensionality of the latent space, but these representations failed to find correlations with the diseases or any comorbidities themselves. Possible reasons for this include the adversarial regression complicating the latent embeddings and the noise in the embeddings presented by the KL divergence term.

Even with the few comparative studies available (see Table S1), is it difficult to establish how challenging these tasks are with the data given. While deep learning studies of AD and MCI detection from brain images are abundant,^3^ comparative disease detection studies of diseases such as epilepsy and Pick's disease are rarer. Even with the difficulties in cross‐study comparisons, a notably poor performance was the multiple sclerosis classification. This strongly suggests that the model trained itself to specialize in emphasizing biomarkers of neurodegenerative disorders rather than the lesions that characterize multiple sclerosis, which was only one of the ten tasks that it was trained on.

An additional complications of this dataset, which is reflective of any real‐world study, are the demographic distributions and the limited means we have of studying them. Sex and age distributions are accurately represented in Table 1, with age being accounted for in a manner similar to our previous work in imbalanced datasets,^38^ but further attempts to gain insights from location or ethnic‐based data are complicated by the messiness of the data. While zip code was unavailable, we compared city names in EHRs to a listing of municipalities and neighborhoods in Massachusetts, which showed that at least 71.7% of the patient cohorts came from Massachusetts. 2543 distinct cities were represented, with 2003 having five or fewer patients in them. The ethnicity data, while it was included in our encoder, was also highly incomplete, consisting only of a question about whether or not the patient identified as Hispanic, and 37% of these answers were blank. When considering just the yes and no answers, 8.6% of the total cohort identified as Hispanic; 11.4% of the non‐dementia group and only 6.2% of the dementia group identified as Hispanic. This is a marked decrease of the American population of 19.1% Hispanic population or the Massachusetts 2020 census that revealed 12% Hispanic population, and considering previous studies showing that dementia rates are higher among Hispanics than non‐Hispanics,^39^ likely represents a bias in who seeks treatment.

The labels used in this project are imperfect and make a clear area of future study. ICD‐10 codes lack the gold standard of medical review that would be necessary for clinical translation of these methods, and these methods can very likely be aided by previous work in using ML methods to simulate medical review for the sake of determining patient diagnosis.^36^ Nonetheless, studies such as the present one are necessary as initial steps. Another potential future direction is prognosis and outcome predictions rather than differential disease detection, which is a more clinically useful task that this framework may easily be adapted to perform.

To comment on practical deployment issues: this project used a significant amount of original code to execute, and it was all on in‐house servers rather than cloud computers. We used a Linux deep learning workstation, with two graphics processing units (GPUs) for the model training, twenty terabytes of storage to store the imaging data, and 128 gigabytes of main memory. Most deep learning frameworks that are commonly used are targeted towards either photographic imaging datasets or large amounts of tabular data that can be accessed with a SQL‐like database; none of those datasets, except for a Pandas data frame to store metadata, were useful for this. The main bottleneck of the model was not the actual training of the neural network, but, rather, the loading and removal of imaging from main memory, which often caused overflow errors until we implemented an original data loader to address the issue. A dedicated, on‐site server was preferred over cloud computing both for data security with patient data and to cut costs. The models typically took 2 to 3 days to train.

Further development on the technical side would likely consist of integrating the outputs of the model into a Clinical Decision Support Report,^40^ which would aid in presenting results to radiologists. A vital next step would, in general, be to study the effects of such results on the decisions of radiologists in clinical practice.

In conclusion, we have presented a unique model that is adapted to the unstructured inputs of real‐world clinical imaging data. We have shown that these deep learning models are capable of cross‐site differential dementia detection, and that more information per patient aids in the end diagnosis. We have also shown that the model, even while accepting an unstructured and skewed dataset, does not overfit to technical confounds, but, rather, focuses on biomarkers that characterize neurodegenerative diseases. This is an essential step forward in utilizing routinely collected clinical imaging data in AI models for patient care.

## CONFLICT OF INTEREST STATEMENT

The authors declare no conflicts of interest.

## CONSENT STATEMENT

Data for this study were accessed under a waiver of informed consent approved by the MGB IRB (Protocol# 2015P001915).

## Supporting information



Supporting Information

Supporting Information
