# Supplementary material for: Differential dementia detection from multimodal brain images in a real‐world dataset
Source: Alzheimers Dement. 2025 Jul 1;21(7):e70362. doi: 10.1002/alz.70362 (PMC12213449; doi:10.1002/alz.70362)
Supplement: Supplementary file 2 — Supporting Information [file ALZ-21-e70362-s001.pdf]

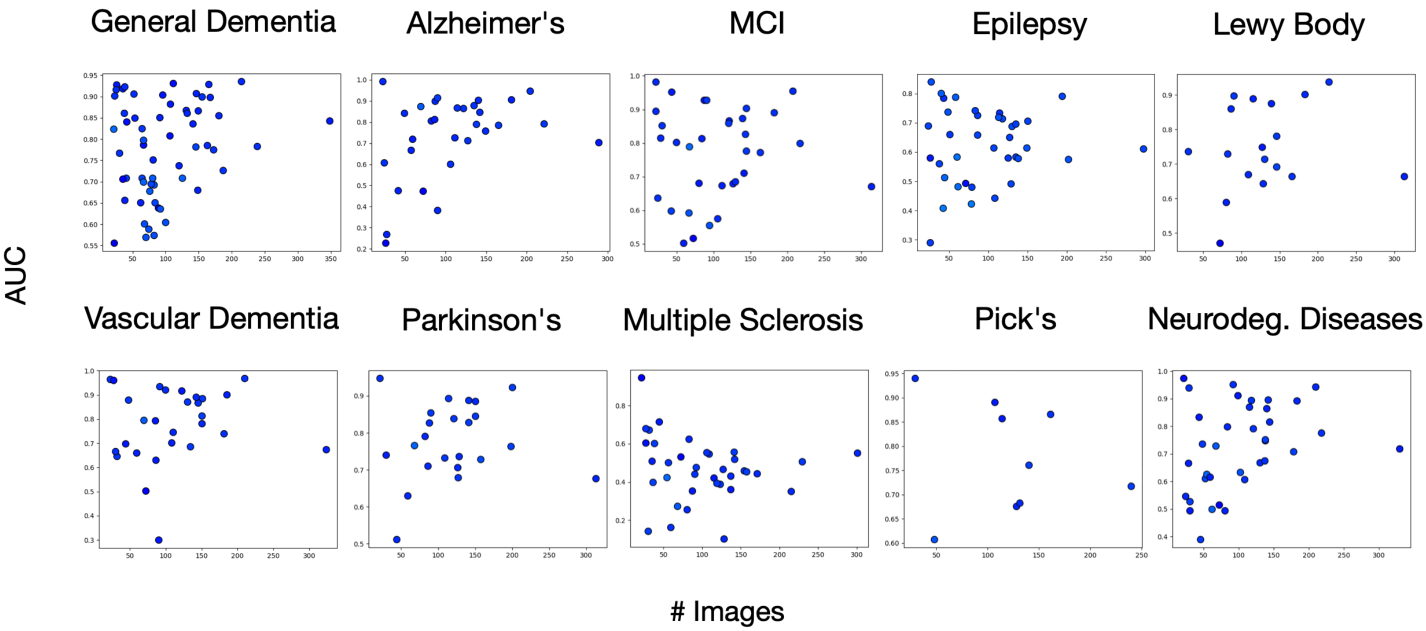

Figure S1. The results on sites with 20 or more images in the disease and non-disease group, as measured by AUC, against the number of images in that site. Each blue dot represents a different external scanning site. This shows approximately the relationship between the number of images in a scanning site and the overall performance of the model on that site, and it complements the graph shown in Figure 4C.

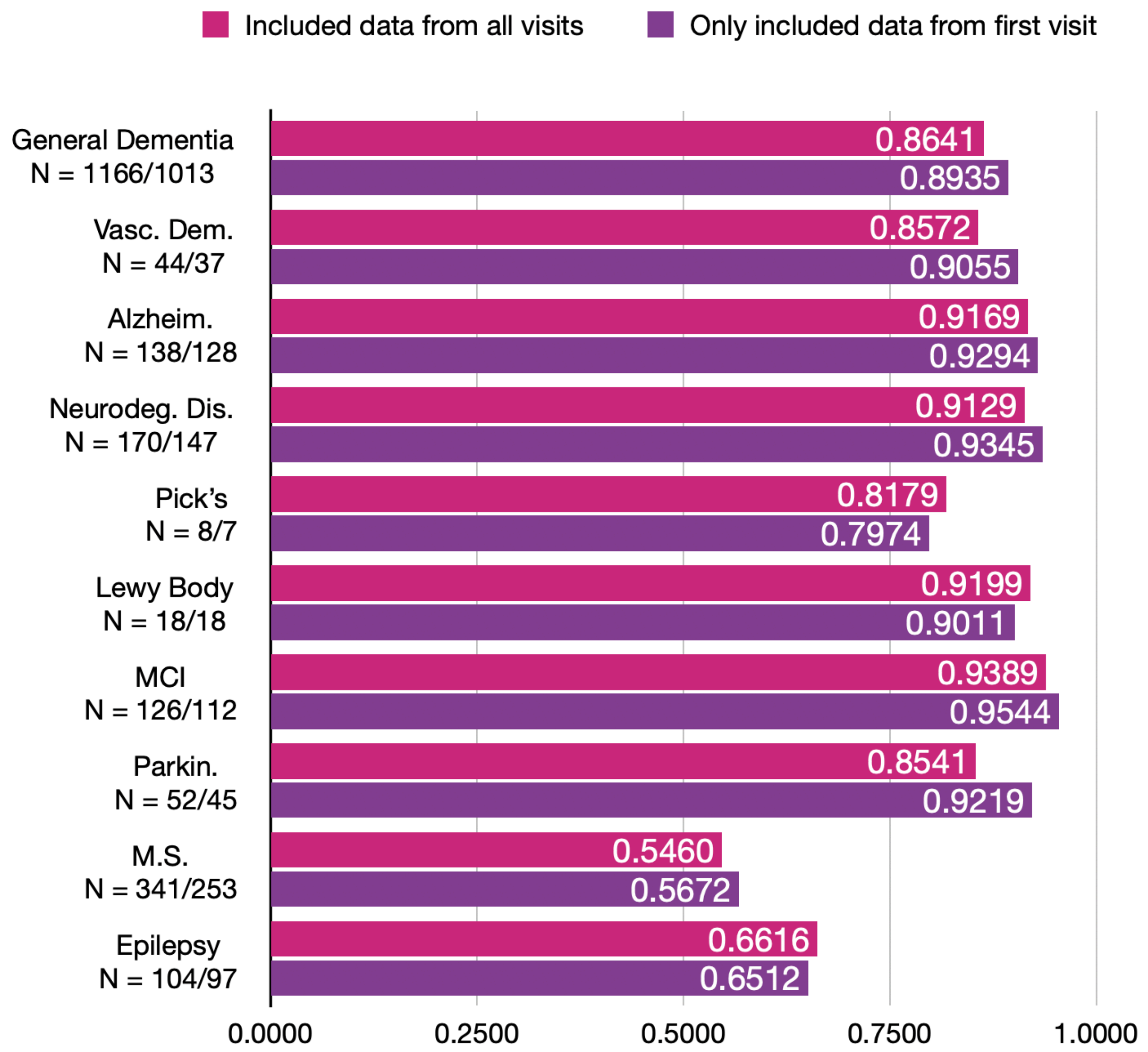

Figure S2. The high-confidence results from all data (presented in Figure 3B) and the subset of those analyses that only included data from the first visit the patient took to the hospital. This shows that the model is capable of making its predictions on an initial screening.

Model performance		Comparative studies				
Disease	AUC	Comparative study	Imaging modalities	AUC	Dataset	N
Vasc. Dem	0.8718	Castellazzi 2020	DTI, fMRI	0.853	Original	33 AD, 27 VD
		Xue et al 2023	Multimodal	0.83	NACC, AIBL, PPMI, NIFD, LBDSU, OASIS and 4RTNI	181 VD, 785 CN
Parkin.	0.8744	Camacho et al 2023	3D T1 MPRAGE	0.87	13 public databanks	1024 PD, 1017 CN
Alzheim.	0.9088	Liu et al 2022	T1 MRI	0.8512	ADNI, NACC	422 AD, 1281 CN
		Kim et al 2022				
		Etminani et al 2022	18FDG-PET	0.964	ADNI	200 AD, 156 CN
Lewy Body	0.9590	Xue et al 2023	Multimodal	0.78	NACC, AIBL, PPMI, NIFD, LBDSU, OASIS and 4RTNI	108 LBD, 858 CN
		Iizuka et al 2019	Brain perfusion SPECT	0.935-0.954	Original	80 LBD
		Etminani et al 2022	18FDG-PET	0.962	Eur LBD consortium	157 LBD, 44 CN
MCI	0.9436	Yang et al 2024	T1 MRI	0.866	ADNI	115 MCI, 133 CN
		Liu et al 2022	T1 MRI	0.6245	ADNI, NACC	322 MCI, 1281 CN
		Etminani et al 2022	18FDG-PET	0.714	ADNI	200 MCI, 156 CN
M.S.	0.5506	Narayana et al 2019	T1 MRI	0.82	Original	519 MS, 1451 CN
		Amini et al 2024	FLAIR MRI	0.90	Original	130 MS
Epilepsy	0.6311	Chang et al 2023	T1 MRI	0.85 (F1)	ADNI, Original	157 EP, 251 CN

Table S1: Comparisons of current model's performance on the ICD classification tasks to a sample of recent imaging studies that have performed similar tasks on brain imaging data. Not all tasks performed in the present study had an analogous outside study to compare to, so those are excluded. Studies that reported AUC were preferred. A comparison to the general dementia task was not offered given that it is an overarching label covering a range of disorders. "Original" datasets indicate data that were acquired specifically for that study.
